# Functional Electrical Stimulation and the Modulation of the Axon Regeneration Program

**DOI:** 10.3389/fcell.2020.00736

**Published:** 2020-08-18

**Authors:** Juan Sebastián Jara, Sydney Agger, Edmund R. Hollis

**Affiliations:** ^1^Burke Neurological Institute, White Plains, NY, United States; ^2^Feil Family Brain and Mind Research Institute, Weill Cornell Medicine, New York, NY, United States

**Keywords:** conditioning injury, regeneration associated genes, sprouting, plasticity, functional recovery, spinal cord injury

## Abstract

Neural injury in mammals often leads to persistent functional deficits as spontaneous repair in the peripheral nervous system (PNS) is often incomplete, while endogenous repair mechanisms in the central nervous system (CNS) are negligible. Peripheral axotomy elicits growth-associated gene programs in sensory and motor neurons that can support reinnervation of peripheral targets given sufficient levels of debris clearance and proximity to nerve targets. In contrast, while damaged CNS circuitry can undergo a limited amount of sprouting and reorganization, this innate plasticity does not re-establish the original connectivity. The utility of novel CNS circuitry will depend on effective connectivity and appropriate training to strengthen these circuits. One method of enhancing novel circuit connectivity is through the use of electrical stimulation, which supports axon growth in both central and peripheral neurons. This review will focus on the effects of CNS and PNS electrical stimulation in activating axon growth-associated gene programs and supporting the recovery of motor and sensory circuits. Electrical stimulation-mediated neuroplasticity represents a therapeutically viable approach to support neural repair and recovery. Development of appropriate clinical strategies employing electrical stimulation will depend upon determining the underlying mechanisms of activity-dependent axon regeneration and the heterogeneity of neuronal subtype responses to stimulation.

## Introduction

Following injury to the adult mammalian central nervous system (CNS), neural circuits are permanently disrupted as severed axons fail to undergo spontaneous regeneration. The limited regenerative response of injured CNS neurons is due to both intrinsic and extrinsic factors. These growth-restrictive mechanisms play a large part in the poor clinical outcomes following brain or spinal cord trauma; however, despite limited regenerative capacity, mounting evidence has shown extensive spontaneous sprouting of CNS axon collaterals in the injured adult CNS.

The mammalian nervous system has an intrinsic capacity for structural and functional reorganization in response to a variety of stimuli during development, learning, or in response to pathological insults (Cramer et al., 2011; von Bernhardi et al., 2017). This innate plasticity is a key component of the system to adapt to a highly changing environment. Injury induced disruption of connectivity in the adult nervous system triggers plastic growth mechanisms and the sprouting of collaterals from spared, intact fibers (Maier and Schwab, 2006). Lesion-induced structural plasticity correlates with a limited degree of function recovery (Chen and Zheng, 2014); however, this innate plasticity falls far short of mediating recovery of damaged CNS circuits.

Recent findings suggest that manipulation of neuronal activity can drive plasticity related growth mechanisms and augment collateral sprouting, thereby enhancing the functional effect of axonal remodeling (Carmel and Martin, 2014). Electrical stimulation has long been known to enhance regeneration of peripheral axons (Hoffman and Binet, 1952; Pockett and Gavin, 1985). More recently, electrical stimulation has been used to enhance CNS plasticity in rodent models as well as to modulate and strengthen spared circuitry in individuals with spinal cord injury. Manipulation of activity-dependent neuroplasticity has great potential for improving neurological recovery from CNS injury. Herein we will discuss the systems-level effects of augmenting activity on injured neuronal circuits.

### Peripheral Nervous System Regeneration

In contrast to most CNS neurons, neurons of the peripheral nervous system (PNS) have a more robust regenerative response to injury (Ramón y Cajal, 1991; Mahar and Cavalli, 2018). This owes to the intrinsic regenerative program activated following PNS injury (McQuarrie and Grafstein, 1973). In the dorsal root ganglia (DRG), this program is characterized by a robust neuronal and non-neuronal response and regulation of a distinct transcriptional program (Tsujino et al., 2000; Costigan et al., 2002; Boeshore et al., 2004; Seijffers et al., 2007; Stam et al., 2007; Chandran et al., 2016). Within the damaged nerve, there is an activation and proliferation of Schwann cells and a robust inflammatory response, resulting in both trophic support and the clearance of myelin debris to allow for axon regeneration (Hall, 1986; Kang and Lichtman, 2013). Peripheral nerve injury has therefore proven to be an invaluable model for studying the underlying mechanisms that support axon regeneration and the structural plasticity of injured neurons.

Despite the innate regenerative potential of PNS neurons after injury, recovery of function remains limited. Several factors can hamper recovery, such as age, extent of injury and disruption of endoneurium, perineurium, or epineurium, and neuroma formation. One critical factor is the slow rate of regeneration in the adult PNS of 1–3 mm per day (Scheib and Hoke, 2013). Axons need to regrow over long distances before reaching appropriate targets, particularly in humans, and a significant delay in target-reinnervation leads to the irreversible atrophy of muscles and end-organs (Lee and Wolfe, 2000). In the absence of appropriate sensory end organs and motor endplate organization, the chances of successful restoration of function dwindle. In order to combat these effects and improve functional outcomes following PNS injury, an ideal treatment would be one that accelerates axon regeneration.

### Peripheral Conditioning and Molecular Pathways That Support Regeneration

The innate regenerative ability of adult mammalian PNS neurons has been used as a model to study the intrinsic mechanisms underlying the regenerative program. Primary sensory neurons are located in the DRG and extend axons into both CNS and PNS. Each axon exhibits a distinct response to injury in the adult. As described above, the peripheral axon retains the ability to regenerate following axotomy. In contrast, the CNS axon of the same cell will fail to regenerate after spinal cord injury. Intriguingly, the regenerative program activated by peripheral nerve injury conditions DRG neurons to mount an enhanced regenerative response to a second injury, whether in the peripheral or central axon (McQuarrie and Grafstein, 1973; Richardson and Issa, 1984). The process of peripheral conditioning results in the activation of a robust signaling cascade, inflammation, and transcriptional changes of thousands of genes in the DRG (Boeshore et al., 2004; Seijffers et al., 2007; Stam et al., 2007). Peripheral conditioning can be driven by axotomy (Richardson and Issa, 1984), inflammation (Steinmetz et al., 2005), demyelination (Hollis et al., 2015b), or electrical stimulation (Senger et al., 2018).

Another important change after conditioning lesion is the transient increase of second messenger cyclic nucleotide cAMP levels. Artificial elevation of cAMP can support a limited amount of sensory axon regeneration in the injured spinal cord (Qiu et al., 2002; Blesch et al., 2012). Downstream modulators of cAMP signaling have been directly linked to the regeneration program, including protein kinase A (Cai et al., 1999, 2001), cAMP response element-binding protein (CREB) (Gao et al., 2004), CREB binding protein (CBP) (Tedeschi et al., 2009) and arginase 1 (Cai et al., 2002).

Activation of the pro-regenerative transcriptional response in the somata of regenerating neurons requires a retrograde signal from the injury site. One candidate for this rapid signal is the early influx of calcium at the injury site (Ziv and Spira, 1995; Wolf et al., 2001; Mandolesi et al., 2004; Ghosh-Roy et al., 2010; Cho et al., 2013). Rapid calcium influx is a hallmark of injury conserved across species from invertebrates to mammals (Rishal and Fainzilber, 2014). Increased calcium levels in the axoplasm act locally on calcium-dependent enzymes related to protein synthesis, cytoskeletal modification, and growth cone formation (Chierzi et al., 2005; Bradke et al., 2012).

Additionally, early axotomy-induced calcium influx rapidly propagates retrogradely to the cell soma. Axotomy of cultured cortical neurons initiates a rapid membrane depolarization at the injury site triggering a fast-retrograde spiking activity and sustained cell body depolarization (Mandolesi et al., 2004). Injury-induced depolarization stimulates the calcium entry through activation of voltage-gated calcium channels (VGCC) or inversion of the sodium-calcium exchange pump following a rise in cytosolic sodium concentration through voltage-gated sodium channels (Mandolesi et al., 2004). Intracellular calcium stores in sensory neurons are released following the initial increase in calcium concentration induced by peripheral injury (Rigaud et al., 2009). These stores are required for the propagation of the calcium wave as pre-emptive depletion of ryanodine receptor-sensitive intracellular calcium stores prevents back propagation and activation at the soma (Cho et al., 2013). It is likely that this calcium wave is a critical preliminary signal to prime neurons for regeneration as preventing it in cortical neurons by inhibiting TTX-sensitive sodium channels impairs neurite extension after *in vitro* injury (Mandolesi et al., 2004). Artificially elevating calcium influx in motor and sensory neurons by activation of the non-selective, light-sensitive cation channel channelrhodopsin can increase the rate of functional regeneration in mice (Ward et al., 2016, 2018). In *C. elegans* sensory neurons, this channelrhodopsin-mediated regeneration has been shown to depend on ryanodine receptor channel release of endoplasmic reticulum calcium stores (Sun et al., 2014). Interestingly, additional epigenetic-mediated changes by calcium increase after injury have been identified. Back propagation of calcium waves regulates epigenetic mechanisms including the release of histone deacetylase 5 (HDAC5) from the nucleus, and inactivation of HDAC3, leading to the initiation of a pro-regenerative transcriptional program (Cho et al., 2013; Hervera et al., 2019).

Electrical stimulation of peripheral nerves elicits this retrograde calcium signal. Genetically encoded calcium indicators can be used to image the calcium wave *in vivo* by fluorescent microscopy. Expression of the calcium indicator GCaMP6s in lumbar level 4 primary sensory neurons has been used to visualize calcium transients in response to sciatic nerve stimulation. Both large and small-diameter sensory somata show maximal calcium responses to low-frequency (20 Hz) stimulation (Chisholm et al., 2018). Short duration pulses (250 ms duration, 250 mA amplitude) preferentially activated large-diameter neurons, while longer duration (1 ms duration, 5 mA amplitude) activated most both A and C fiber cell bodies. Whether these distinct stimulation parameters elicit differential cellular responses is unknown.

The spatiotemporal regulation of calcium influx can regulate the regenerative program as calcium transients in the proximal segment after injury impair regeneration. In non-regenerating sensory neurons of drosophila, injury induces local calcium transients through the mechanosensitive cation channel dmPiezo, which inhibits axon regeneration by activating the calcium signaling regulator Ca^2+^/calmodulin-dependent protein kinase II (CamKII) (Song et al., 2019). Nitric oxide synthase (NOS) and the cGMP-dependent kinase PKG act downstream of Piezo-mediated calcium signaling to inhibit axon regeneration in drosophila (Song et al., 2019). Piezo-mediated inhibition of regeneration is conserved in mouse models of sensory axotomy and conditional deletion of *Piezo1* enhances regeneration *in vivo* (Song et al., 2019). Furthermore, expression of the α2δ2 subunit of voltage gated calcium channels has been proposed to be a developmental switch underlying the decrease in axonal growth capacity that accompanies PNS maturation (Tedeschi et al., 2016). Genetic deletion of the encoding gene *Cacna2d2* promotes neurite elongation from cultured primary sensory neurons, while α2δ2 overexpression inhibits elongation through Cav2-mediated influx of pre-synaptic extracellular calcium (Tedeschi et al., 2016). Blockade of α2δ2 by clinically approved gabapentinoid drugs promotes neurite elongation *in vitro* and sensory axon regeneration and regenerative sprouting from injured corticospinal axons *in vivo* (Tedeschi et al., 2016; Sun et al., 2020).

### Neuronal Activity and Molecular Control Over Axon Growth

Neuronal activity can activate growth-associated molecular pathways. In an optic nerve model of CNS injury, driving activity in retinal ganglion cells (RGCs) by high-contrast visual stimulation or via chemogenetics approaches can enhance optic nerve regeneration (Lim et al., 2016). In contrast, suppressing neuronal activity with chemogenetics prevents the pro-regenerative effect of high-contrast visual stimulation (Lim et al., 2016). Furthermore, light-sensitive RGCs that express melanopsin are resistant to axotomy and exhibit high levels of phosphorylated ribosomal protein S6 (pS6) (Li et al., 2016). RGC pS6 levels decrease after axon elongation during development and this downregulation can be attenuated by deletion of the phosphatase and tensin homolog (PTEN) (Park et al., 2008). *PTEN* deletion from RGCs and other CNS neurons leads to elevated PI3K/mTOR (mammalian target of rapamycin) signaling, enhanced phosphorylation of S6, and an increased capacity for axonal growth after injury (Park et al., 2008; Liu et al., 2010). Alternatively, activating mTOR via over-expression of a constitutively active form of ras homolog enriched in brain 1 (cRheb1) appears to enhance RGC regeneration, though not nearly as robustly as *PTEN* deletion (Park et al., 2008; Lim et al., 2016).

Chronic electrical stimulation of the motor cortex over ten consecutive days leads to activation of the mTOR pathway, inactivation of PTEN, and increased phosphorylation of ribosomal protein S6 (Zareen et al., 2018). Additionally, this chronic stimulation drives increased levels of Janus kinase-signal transducer and activator of transcription (JAK/STAT) signaling (Zareen et al., 2018), a critical mediator of cytokine signaling. Enhancing JAK/STAT signaling through deletion of *suppressor of cytokine signaling 3* (*SOCS3*) increases optic nerve regeneration (Smith et al., 2009). JAK/STAT signaling acts independently of mTOR, while *SOCS3* and *PTEN* co-deletion deletion act synergistically to promote sustained optic nerve regeneration (Sun et al., 2011). In the motor cortex, the activation of these separate molecular pathways by chronic, daily electrical stimulation drives distinct aspects of structural remodeling in the intact corticospinal circuitry. New corticospinal collaterals sprout and form synaptic connections in the spinal cord during 10 days of stimulation. Pharmacological studies implicate mTOR signaling in collateral formation as corticospinal collateral sprouting is blocked by rapamycin; whereas, inhibiting Stat3 activation with AG490 reduced both bouton-like structures on corticospinal axons as well as levels of cFos expression in ipsilateral cervical spinal cord neurons without affecting corticospinal collateral formation (Zareen et al., 2018). These studies demonstrate that not only can electrical stimulation activate growth-promoting molecular pathways, it can strengthen the formation of novel connections needed to elicit functional recovery.

Regeneration competent PNS neurons have been extensively studied to determine the molecular pathways critical for driving axon regeneration. Regeneration-associated genes (RAGs) have been largely defined as the coordinated and complementary genes activated by peripheral conditioning paradigms in PNS neurons. These include developmental growth-associated proteins (e.g., GAP-43, CAP23, and SPRR1A), transcription factors (e.g., ATF-3, c-Jun, Sox11, Smad1, Klf family members, and Stat3), and signaling pathways (MAPK, cytokine, JAK-STAT, TGF-b, neurotrophin) (Chandran et al., 2016; Mahar and Cavalli, 2018). In much the same manner that electrical stimulation of the cortex activates pro-regenerative molecular pathways, electrical stimulation of peripheral nerves has been shown to induce identified RAG expression in both sensory and motor neurons. Direct low-frequency electrical stimulation of the intact rodent sciatic nerves at 20 Hz for 1 h induces a significant upregulation in the expression of growth associated protein-43 (GAP-43), the neurotrophin BDNF and its high-affinity receptor trkB, as well as increased phosphorylation of the transcription factor cAMP response element binding protein (CREB) in DRGs neurons (English et al., 2007; Senger et al., 2018, 2019). This gene induction is comparable to injury-induced RAG induction (Senger et al., 2018, 2019). Similarly, brief electrical stimulation of the injured rat femoral nerve rapidly activates a RAGs response in motor neurons with upregulation of GAP43 and Tα1-Tubulin, as well as an increase in BDNF and trkB expression in stimulated motor neurons (Al-Majed et al., 2000a, 2004). The effects of electrical stimulation extend to perineuronal glial cells, with increased GFAP expression in satellite glia (Senger et al., 2018). In addition to the transcriptional regulation that occurs with electrical stimulation, 1 h of low-frequency stimulation of intact rat sciatic nerves increases intracellular cAMP levels in DRG neurons similar to nerve injury (Udina et al., 2008). Despite the fact that electrical stimulation and axotomy induce similar increases in cAMP, 1 h of low-frequency electrical stimulation is not sufficient to fully recapitulate the regenerative response of peripheral conditioning of sensory neurons to a central spinal cord injury (Udina et al., 2008; Goganau et al., 2018).

While electrical stimulation activates many of the same molecular pathways as peripheral conditioning via crush injury, it does not fully recapitulate the growth-promoting effects of conditioning. Following a subsequent peripheral injury, previous exposure to low-frequency electrical stimulation enhances the initiation of regeneration, but does not increase the rate of peripheral motor or sensory axon regeneration (Al-Majed et al., 2000b; Brushart et al., 2002). The limited effectiveness of electrical conditioning extends to the CNS as well, as regeneration of the central axon of dorsal column projecting sensory neurons after a spinal cord injury is less robust in rats conditioned 1 week prior with electrical stimulation than those subjected to nerve crush injury (Goganau et al., 2018). Electrical stimulation immediately following a dorsal column spinal cord injury allows for an increased initiation of regeneration; however, these axons fail to elongate through the injury site, in contrast to more robust regenerative effects of nerve crush injury (Udina et al., 2008). Despite the limitations of electrical stimulation compared to nerve crush, stimulation is a more attractive potential therapeutic approach to enhancing axon regeneration. The use of a conditioning nerve crush injury in patients is impractical as it would increase the risk of surgical complications and nerve crush elicits neuropathic pain (Hollis et al., 2015b).

Some of the limited effectiveness of current electrical conditioning may be alleviated by further optimization of stimulation parameters. Primary sensory neurons are sensitive to the frequency of electrically stimulated action potential patterns, activating discrete transcriptional programs based upon temporal changes in bursting patterns (Lee et al., 2017). It has long been known that specific patterns of induced neural activity in primary sensory neurons regulate immediate early gene expression independent of intracellular calcium levels (Sheng et al., 1993). The duration of neural activity induces unique gene expression profiles in a mechanistically distinct manner (Tyssowski et al., 2018). Defined action potential bursts underlie a temporal specificity in calcium influx through voltage gated calcium channels, leading to a tightly regulated activation of cAMP-responsive element binding protein (CREB) and mitogen-activated protein kinase (MAPK) signaling pathways (Fields et al., 1997; Wheeler et al., 2012). These same mechanisms may regulate the regenerative responses to discrete patterns of electrical stimulation. Indeed, modulation of stimulation frequency and current appears to alter the morphological and electrical properties of regenerated rat sciatic nerve (Lu et al., 2008, 2009). Despite the activation of the regenerative program found *in vivo* by low frequency (20 Hz) electrical stimulation, 20 Hz trains separated by 5 min intervals arrested neurite outgrowth of primary sensory neurons *in vitro* (Enes et al., 2010). Calcium influx through voltage-gated Cav1.2 calcium channels was proposed to mediate the stimulation evoked arrest of axon growth, with enhanced neurite outgrowth from cultured Cav1.2 deleted sensory neurons (Enes et al., 2010). Stimulation intensity of primary sensory neurons differentially regulates signaling through Cav1 and Cav2 channels (Wheeler et al., 2012), potentially driving distinct transcriptional responses and differences in the regenerative response.

Furthermore, maintenance of the energy supply in neurons is critical to meet metabolic demands and supporting physiological homeostasis. Axonal mitochondrial transport is crucial to this end. Following injury, the bioenergetic balance is highly disrupted, with mitochondrial depolarization and ATP depletion (Zhou et al., 2016). Increasing mitochondrial transport enhances peripheral and central axon regeneration (Cartoni et al., 2016; Zhou et al., 2016; Han et al., 2020). Electrical stimulation of peripheral nerves increases mitochondrial transport in a frequency dependent fashion (Sajic et al., 2013). Low frequency stimulation (1 Hz) mobilizes mitochondria transport in both anterograde and retrograde directions, while higher frequency (50 Hz) stimulation further augmented anterograde transport only (Sajic et al., 2013). The increase in mitochondrial transport mediated by electrical stimulation may support the regenerative response following injury.

### Functional Effects of Electrical Stimulation on Circuit Recovery

**FIGURE 1:**
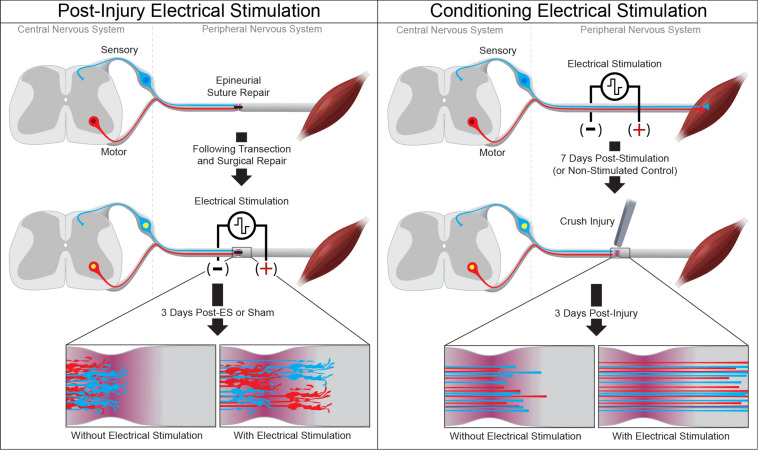
Electrical stimulation enhances peripheral axon regeneration. A single session of low-frequency electrical stimulation (1 h, 20 Hz) can enhance motor and sensory axon regeneration following epineurial suture repair **(left)** or when used as a conditioning stimulus prior to subsequent axotomy **(right)**.

Electrical stimulation has been used to hasten the recovery of both peripheral and central circuits after injury. While limited in comparison to the growth-promoting effects of conditioning crush injury, post-injury electrical stimulation has proven safe and effective in both animal models and humans. Low-frequency (4 Hz) stimulation of the soleus muscle in the rabbit was used to shorten the time for recovery of soleus function after axonotmesis of the soleus motor nerve (Nix and Hopf, 1983). This more rapid recovery of function elicited by post-injury electrical stimulation has also been observed in rodent models of nerve repair. A single hour of low-frequency electrical stimulation immediately following sciatic nerve transection and repair enhances axon regeneration and accelerates recovery of motor and sensory function, as measured by electrophysiological recordings and thermal sensitivity in the reinnervated hindpaw (Figure 1; Singh et al., 2012). Similar single 20 Hz electrical stimulation has been shown to enhance recovery after femoral nerve transection and epineurial suture repair, with quadriceps function returning 6-weeks earlier in stimulated mice than in controls (Ahlborn et al., 2007). Low-frequency stimulation post-surgery has been combined with daily locomotor training to accelerate the recovery of neurophysiological function after sciatic transection and epineurial suture repair (Asensio-Pinilla et al., 2009). More recently, the use of conditioning electrical stimulation was directly compared to nerve crush conditioning in a rat model of tibial nerve repair, in which the tibial nerve was conditioned 1 week prior to a nerve transection and epineurial suture repair (Senger et al., 2019). In these studies, conditioning by electrical stimulation was found to elicit greater physiological and functional recovery than conditioning by nerve crush. This apparent discrepancy with the effects observed *in vitro* and in models of subsequent spinal cord injury (Goganau et al., 2018) is likely due to slowing of the regenerating axons upon reaching the disrupted cytoarchitecture, proliferative Schwann cells, and inflammation at the nerve crush site. The efficacy of both pre- and post-injury electrical stimulation in enhancing regeneration of rodent peripheral nerves and accelerating functional outcomes, together with the feasibility of its use in bedside settings makes brief low frequency electrical stimulation a clinically relevant approach to mediate PNS repair. Indeed, clinical studies have found that electrical stimulation following surgical intervention to treat either full digital nerve transection or median nerve crush (carpal tunnel syndrome) is well-tolerated and enhances the recovery of both sensory and motor function (Gordon et al., 2010; Wong et al., 2015).

**FIGURE 2:**
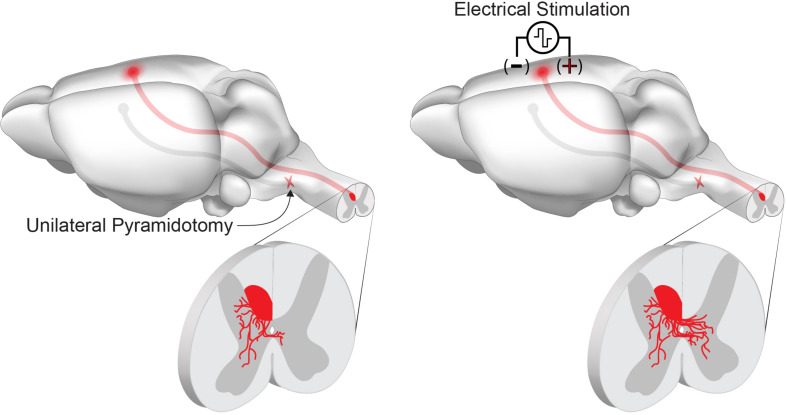
Chronic electrical stimulation can drive corticospinal tract sprouting following injury. Unilateral pyramidotomy activates spontaneous collateral sprouting from the contralateral, intact corticospinal tract within the spinal cord. Chronic stimulation of the motor cortex activates neuron intrinsic growth pathways and enhances collateral sprouting and connectivity of spared corticospinal tract axons.

Within the CNS, chronic electrical stimulation drives collateral sprouting from intact descending corticospinal circuits (Figure 2). The corticospinal circuit is the primary motor pathway in primates and largely projects to the contralateral spinal cord. Ten days of stimulation (333 Hz, 45 ms bursts, every 2 s, for 6 h/day) of the medullary pyramid promotes sprouting of the corticospinal tract across the midline into the ipsilateral, denervated spinal cord (Brus-Ramer et al., 2007). This collateral sprouting is similar to what is observed following unilateral transection of the medullary pyramids (pyramidotomy), when the absence of corticospinal input to the spinal cord leads to cross-midline sprouting from the spared, intact corticospinal tract (Brus-Ramer et al., 2007; Lee et al., 2010). Performing chronic electrical stimulation after unilateral pyramidotomy results in an additive effect on corticospinal collateral sprouting (Brus-Ramer et al., 2007). Stimulation of the contralesional motor cortex mimics the direct stimulation of the pyramids, and this enhanced circuit plasticity drives recovery of skilled locomotor function after unilateral pyramidotomy in acute and chronic injury models (Carmel et al., 2010, 2014). Recovery is likely mediated by the formation of a novel, ipsilateral corticospinal circuit as inactivation of both ipsilateral and contralateral motor cortex impairs the behavioral recovery, while intact animals are only affected by contralateral cortical inactivation (Carmel et al., 2014). The growth-promoting effects of cortical stimulation are not restricted to intact circuits, as cortical electrical stimulation following cervical spinal cord injury drives corticospinal axon growth proximal to the injury site and enhances the recovery of forelimb function (Batty et al., 2020). In addition to the enhancement of axonal growth, the restoration of function through novel corticospinal circuits requires the synapse-promoting effects of cortical electrical stimulation. Pairing of cortical stimulation with peripheral afferents transiently strengthens the response to descending corticospinal input in acute experiments in rats (Mishra et al., 2017). Similar results have been demonstrated in healthy and spinal cord injured individuals (Bunday and Perez, 2012). In rats, repeated pairing of intermittent theta burst cortical stimulation with *trans-*spinal direct current stimulation after cervical spinal cord injury results in a strengthening of novel connections in the spinal cord. Furthermore, this paired stimulation paradigm can support the recovery of dextereous forelimb movements that depend on corticospinal function (Zareen et al., 2017; Yang et al., 2019). A brief summary of rodent electrical stimulation studies can be found in Table 1.

**TABLE 1:** A brief summary of rodent electrical stimulation studies in the PNS and CNS.

References	CNS/PNS	Stimulation substrate	Parameters	Effects
Al-Majed et al., 2000a	PNS	Electrode wired around injured rat femoral nerve	20 Hz, 0.1 ms, 3 V, continous 1-h	Increased BDNF and trkB expression in motor neurons at 8 h and 2 days after ES.
Al-Majed et al., 2000b	PNS	Electrodes wired around injured rat femoral nerve	20 Hz, 0.1 ms, 3 V, continous 1-h	ES enhanced initiation of motor neuron regeneration after femoral nerve repair.
Al-Majed et al., 2004	PNS	Electrode wired around injured rat femoral nerve	20 Hz, 0.1 ms, 3 V, continous 1-h	Upregulation of GAP43 and Tα1-Tubulin expression in motor neurons at 2days after ES.
English et al., 2007	PNS	Cuff electrode around injured mouse common fibular nerve	20 Hz, 0.1 ms, 0.5 to 5 V (2× motor threshold), continuous 1-h	Enhanced YFP+ axon regeneration through nerve allograft in a NT-4/5 dependent manner. Increased the levels of BDNF and trkB in injured and intact DRGs at 7 and 14 days after ES.
Geremia et al., 2007	PNS	Stainless steel wire electrodes on opposite sides of injured femoral nerve	20 Hz, 0.1 ms, 2× motor threshold 1-h	Increased sensory neuron regeneration after femoral nerve trunk repair, increased BDNF and GAP-43 expression in DRG neurons.
Udina et al., 2008	PNS	Electrode wired around intact rat sciatic nerve	20 Hz, 0.02 ms, ∼3 to 5 V (2× motor threshold), continous 1-h	ES enhanced central axon regeneration was less robust than nerve crush effects after SCI. Increased cAMP levels in DRGs 7 days after ES, comparable to nerve injury.
Chisholm et al., 2018	PNS	Cuff electrodes surrounding intact mouse sciatic nerve	1, 2, 5, and 20 Hz, ES-1: 250 μA, 250 μs. ES-2: 5 mA, 1 ms	Intensity- and frequency-dependent rapid calcium increase in DRG neurons
Goganau et al., 2018	PNS	Cuff electrodes around intact rat sciatic nerve	20 Hz, 0.2 ms, 2× motor threshold, continous 1-h (ES1), 7-h (ES2), or 1-h daily per 7 days (ES3).	ES enhanced central axon regeneration was less robust than nerve crush effects after SCI. 1 h of ES per day for 7 days and 7 hours of ES followed by 7 days of waiting resulted in similar *in vitro* neurite outgrowth.
Senger et al., 2018	PNS	Electrode wired around intact rat common peroneal nerve	20 Hz, 0.1 ms, continuos 1-h	Upregulation of GAP-43 and BDNF expression in DRG neurons and GFAP in satellite cells at 3 days after ES. Similar to injury-induced changes.
Senger et al., 2019	PNS	Electrode wired around intact rat tibial nerve	20 Hz, 0.1 ms, continuos 1-h	Increased pCREB levels in DRGs at 3 days after ES. 7 day prior electrical conditioning enhanced regeneration and reinnervation.
Brus-Ramer et al., 2007	CNS	Tripolar electrodes on the surface of rats pyramidal tract	333 Hz, 45 ms burst every 2 s, 6 h daily per 10 days.	Chronic ES enhanced intact corticospinal collateral sprouting.
Carmel et al., 2014	CNS	Implanted electrodes into rat forelimb motor cortex	333 Hz, 45 ms burst every 2 s, 6 h daily per 10 days	Chronic ES induced recovery of skilled locomotor function.
Zareen et al., 2017	CNS	Combined bilateral rat epidural (M1) iTBS, and spinal cord (C4-T2) tsDCS stimulation 7 weeks after SCI	iTBS: stimulation intensity 75% of motor threshold. tsDCS: 1.5 mA for 2.5 s and returned to 0 over 2.5 s. Combined stimulation for 30 min daily per 10 day.	Combined ES enhanced injury-dependent corticospinal collateral sprouting below and above the level of injury, and enhanced skilled forelimb functional recovery.
Zareen et al., 2018	CNS	Implanted electrodes into rat forelimb motor cortex	333 Hz, 0.2 ms duration every 2 s, 1.1 to 2 mA, continous 6 h daily per 10 days	Chronic ES induced activation of mTOR and Jak/STAT pathways, inactivation of PTEN, and increased phosphorylation of ribosomal protein s6. Chronic ES induced mTOR-dependent collateral sprouting of intact corticospinal tract.
Jack et al., 2018	CNS	Implanted electrodes into rat forelimb motor cortex	333 Hz, 0.2 ms duration every 500 ms, 30 pulses per train	Single ES to injured CST increased collateral sprouting above injury site, with no further improvement in recovery of function.
Batty et al., 2020	CNS	implanted electrodes into rats forelimb motor cortex	333 Hz, 30 pulses of 0.2 ms width every 0.5 s, for 30 min	Single ES to intact CST enhanced corticospinal collateral sprouting above SCI and improved motor function recovery.

### Therapeutic Potential of Electrical Stimulation

In contrast to the robust regenerative response in rodents, injury of peripheral nerves in humans often results in incomplete or inadequate functional recovery (Scheib and Hoke, 2013). The efficacy of pre- and post-injury electrical stimulation in improving regeneration and functional outcomes has been demonstrated in multiple rodent models of peripheral nerve injury. This, combined with the feasibility of use in bedside settings, makes brief, low-frequency electrical stimulation a clinically viable approach in peripheral nerve repair as a means to mitigate the effects of slow regeneration rate on the loss of end-organs and muscle atrophy (Lee and Wolfe, 2000). To date, 1 h of low-frequency (20 Hz) electrical stimulation has been successfully applied in clinical practice (Gordon et al., 2010; Wong et al., 2015). Individuals with motor axon loss from chronic median nerve compression at the wrist underwent decompression surgery. The group treated with single low-frequency electrical stimulation for 1 h after surgery showed enhanced reinnervation of thenar muscles characterized by improved motor and sensory nerve conduction in comparison to the surgery only group (Gordon et al., 2010). Electrical stimulation was also effective in improving physiological recovery and the return of normal sensory function when applied following epineurial repair of transected digital nerves (Wong et al., 2015). The effectiveness of these clinical findings on peripheral nerve repair are consistent with the evidence obtained from animal models. The recent findings that pre-conditioning of peripheral nerves with electrical stimulation has a robust effect on axon regeneration (Senger et al., 2019) suggests that there may be a benefit to performing pre-operative electrical stimulation in cases of surgical interventions involving intact nerves, such as nerve transfer. Further clinical studies are warranted to determine if the functional benefit of low-frequency electrical stimulation translates to long-distance nerve repair surgeries, such as brachial plexus repair. Additionally, the potential for peripheral nerve stimulation to enhance the sprouting of central sensory axons after spinal cord injury could be used to restore sensory function critical for movement (Takeoka et al., 2014). Strengthening ascending mechanosensory input to only a small proportion (<5%) of dorsal column nuclei is sufficient to support some level of functional recovery (Hollis et al., 2015a), so the translation of PNS electrical stimulation may prove to be a viable approach to restore sensory function after spinal cord injury.

Despite the limited regenerative capacity of CNS axons after spinal cord injury, some spontaneous compensatory sprouting from spared and injured axons can occur in both rodent and non-human primates (Ghosh et al., 2010; Rosenzweig et al., 2010). Mounting evidence indicates that this post-lesion plasticity can be increased by artificially driving neuronal activity. Epidural electrical stimulation has been employed in both animal models and in paraplegic individuals to engage motor circuits and support locomotor recovery. The extent to which this neuromodulation can have lasting impacts on motor circuit remodeling after injury is not yet known. Following complete thoracic spinal cord injury in rats, spinal motor networks are engaged during periods of epidural electrical stimulation, which allows for locomotion in the absence of descending input (Ichiyama et al., 2005). Chronically injured paraplegic individuals have similarly shown engagement of spinal motor circuits in the presence of epidural electric stimulation. Epidural stimulation in motor complete individuals allows for volitional movement of paralyzed leg muscles, including overground walking during stimulation (Harkema et al., 2011; Angeli et al., 2014, 2018). Furthermore, repeated pairing of activity-based training with epidural stimulation in a motor complete individual over several years has supported the recovery of trained volitional motor movements, independent of stimulation (Rejc et al., 2017). Patterning of epidural stimulation over the lumbar spinal cord to sequentially activate agonist-antagonist muscle groups supports the recovery of stimulation-mediated walking in chronic paraplegic individuals when paired with intensive rehabilitation (Wagner et al., 2018). Furthermore, some of the individuals enrolled in this study regained voluntary leg movements in the absence of stimulation. The underlying activity-dependent mechanism activated by epidural electrical stimulation paired with rehabilitation remains unknown. It is likely that the initial responses are activating motor circuits below the injury and residual supraspinal circuitry is able to engage these excited motor networks either directly or indirectly through spared propriospinal circuitry. The eventual recovery of limited volitional control in several individuals may result from engaging many of the same neuroplasticity mechanisms identified in electrical stimulation studies in animal models.

In contrast to implanted epidural stimulators, transcutaneous electrical spinal cord stimulation (TESS) is a non-invasive and painless neuromodulation strategy that augments motor and sensory function after SCI (Gerasimenko et al., 2015; Hofstoetter et al., 2015; Gad et al., 2018; Benavides et al., 2020). Cervical TESS, in combination with training, has been shown to induce a long-term improvement in volitional motor control and restoration of hand sensory function individuals with chronic incomplete SCI (Inanici et al., 2018). TESS utilizes currents from 5 to 50 Hz, with a carrier frequency (Russian current) between 5 and 10 kHz (Benavides et al., 2020). Changes in spinal sensory and motor circuit excitability have been proposed to underlie TESS-mediated functional effects, with neuroplasticity mechanisms facilitating a reorganization of spinal networks during intensive, rehabilitative training (Inanici et al., 2018; Benavides et al., 2020). The extent of anatomical circuit remodeling induced by TESS remains to be determined.

Among the different approaches that have been proposed to enhance post-lesion plasticity, extrinsic manipulation of neuronal activity by electrical stimulation is an attractive therapeutic approach. Electrical stimulation has been demonstrated to engage plasticity mechanisms in several central and peripheral neural circuits and has already shown feasibility in clinical settings. Development of appropriate strategies will likely depend upon the selective activation of desired neural subtypes in a temporally and spatially organized manner. Non-targeted electrical fields have been used to trigger population responses; however, it may be that distinct electrical stimulation parameters can selectively affect neuronal subtype responses and circuit-specific functional outcomes. Whether the molecular mechanisms activated by electrical stimulation are consistent across neuronal populations is unknown. The sprouting response of corticospinal axons to electrical stimulation contrasts with the elongation observed in stimulated PNS axons. These differences may arise from disparate stimulation parameters, discrete responses of these distinct populations to electrical stimulation, or from interactions of conserved stimulation-mediated molecular pathways with the intrinsic limitations of adult CNS neurons to axon elongation. Further studies will be required to identify whether CNS-tuned parameters of electrical stimulation can drive a regenerative response in transected corticospinal axons.

In the context of SCI, preclinical and clinical studies have clearly demonstrated that the stimulation of local spinal networks can drive lasting functional improvements (Ievins and Moritz, 2017; Courtine and Sofroniew, 2019; Hutson and Di Giovanni, 2019). Cortical and spinal cord paired electrical stimulation is an attractive approach to both enhance intrinsic sprouting and strengthen residual and newly formed collateral connections within the spinal cord (Dixon et al., 2016; Mishra et al., 2017). Additionally, it is likely that targeted rehabilitation or concurrent therapies, such as constraint-induced movement therapy, will be needed to strengthen and engage novel circuitry driven by electrical stimulation. The development of novel technologies will allow for the combining of biomedical devices for stimulation with rehabilitation and molecular or genetic control over neuroplasticity to support recovery after neurological injury and improve individuals’ quality of life.

## Author Contributions

JJ and EH wrote and edited the manuscript. SA created the illustrations. All authors contributed to the article and approved the submitted version.

## Conflict of Interest

The authors declare that the research was conducted in the absence of any commercial or financial relationships that could be construed as a potential conflict of interest.
